# Opposing effects on the cell cycle of T lymphocytes by Fbxo7 via Cdk6 and p27

**DOI:** 10.1007/s00018-016-2427-3

**Published:** 2016-12-03

**Authors:** Shachi P. Patel, Suzanne J. Randle, Sarah Gibbs, Anne Cooke, Heike Laman

**Affiliations:** Department of Pathology, University of Cambridge, Cambridge, UK

**Keywords:** Fbxo7, T-cell development, Cell cycle, Cdk6, p27

## Abstract

G_1_ phase cell cycle proteins, such as cyclin-dependent kinase 6 (Cdk6) and its activating partners, the D-type cyclins, are important regulators of T-cell development and function. An F-box protein, called F-box only protein 7 (Fbxo7), acts as a cell cycle regulator by enhancing cyclin D-Cdk6 complex formation and stabilising levels of p27, a cyclin-dependent kinase inhibitor. We generated a murine model of reduced Fbxo7 expression to test its physiological role in multiple tissues and found that these mice displayed a pronounced thymic hypoplasia. Further analysis revealed that Fbxo7 differentially affected proliferation and apoptosis of thymocytes at various stages of differentiation in the thymus and also mature T-cell function and proliferation in the periphery. Paradoxically, Fbxo7-deficient immature thymocytes failed to undergo expansion in the thymus due to a lack of Cdk6 activity, while mature T cells showed enhanced proliferative capacity upon T-cell receptor engagement due to reduced p27 levels. Our studies reveal differential cell cycle regulation by Fbxo7 at different stages in T-cell development.

**Electronic supplementary material:**

The online version of this article (doi:10.1007/s00018-016-2427-3) contains supplementary material, which is available to authorized users.

## Introduction

Fbxo7 (F-box only protein 7) is a multi-functional protein with remarkable tissue-specific effects and is of clinical relevance in a variety of human pathologies, ranging from Parkinson’s disease and blood disorders to cancer [1–3]. Fbxo7 is a member of the 69 F-box domain-containing protein family, which function as substrate-docking subunits of SCF (Skp1-Cullin1-F-box)-type E3 ubiquitin ligases [4–8]. These ligases act at the last transference step in the modification of protein substrates by ubiquitin. Substrates of SCF^Fbxo7^ ligase include the kinetochore protein HURP, NF-κB signalling regulators c-IAP and TRAF2, and NRAGE, a protein involved in apoptosis arising from nerve growth factor signalling [9–12]. In addition to this canonical function, Fbxo7 has other well-documented SCF-independent roles, including acting as a cell cycle regulator in two ways: first, by interacting directly with the G1 phase kinase Cdk6, promoting its binding activation by D-type cyclins, and second, by binding and stabilising the levels of cyclin-dependent kinase inhibitor (CDKI), p27 [1, 13, 14]. This cell cycle regulatory role of Fbxo7 is important in erythropoiesis, and we have reported that the reduction of Fbxo7 in a mouse causes anaemia, caused by a failure of differentiating erythroblasts to withdraw from the cell cycle due to insufficient levels of p27 [15, 16]. Multiple GWAS studies reinforce the importance of Fbxo7 in red blood cell (RBC) biology as SNPs in *FBXO7* are associated with clinically relevant RBC parameters [17–20]. In addition to GWAS studies of the blood, similar studies on families with pedigrees showing cases of the early onset Parkinson’s disease revealed the homozygous inheritance of point mutations in *FBXO7* to be causative [21–23]. Subsequently, also named PARK15, Fbxo7 was found to interact directly with two other genes mutated in Parkinson’s disease, PINK1/PARK6, and Parkin/PARK2, to promote mitophagy [24]. Pathogenic point mutations map to functional domains in Fbxo7 including T22M within its N-terminal ubiquitin-like (Ubl) domain that interacts directly with Parkin; R378G adjacent to the F-box domain, which reduces its ability to form an E3 ligase complex; and R498X within one of its substrate-recruiting domains near the end of the protein [3]. Collectively, these mutations point to multiple defects in Fbxo7’s many functions as contributing to neurodegeneration. However, as neurons are post-mitotic, this is unlikely to involve its cell cycle regulatory activity.

In addition to its cell cycle regulatory function in erythropoiesis, we reported that Fbxo7 has an anti-proliferative function and a role in promoting the maturation of precursor B lymphocytes, caused by stabilising p27 levels and inhibiting S phase kinase activity [16]. G1 phase cell cycle proteins are known to play key roles in regulating proliferation and maturation of T lymphocytes in the thymus. Two of the three D-type cyclins are strongly expressed, cyclin D2 before the rearrangement of T-cell receptor (TCR) β, and cyclin D3 afterwards. These cyclins appear to act primarily through activation of Cdk6, rather than Cdk4. In support of its non-redundant role, Cdk6 knock-out mice have a striking reduction in thymus size and show a block in differentiation at the DN3 stage along with impaired proliferation at the DN2 and DN3 stages [25, 26]. Cyclin D3 null mice also have a small thymus, due to deficient expansion of immature thymocytes at the DN4 stage [27]. Despite cyclin D2 being highly expressed at DN1 to DN3 stages, it is dispensable for T-cell differentiation as cyclin D2 knock-out mice do not show thymic defects, which the authors of that study attributed to compensation by cyclin D3 [28]. Cyclin D3 and Cdk6 are both proto-oncogenes in T cells, and are overexpressed in T-cell malignancies, like T-ALL and T-cell lymphoma [27]. Moreover, they are thought to function as critical downstream transducers of other oncogenic signalling pathways, like Notch and p65^Lck^. We previously reported that the over-expression of Fbxo7 causes a late-onset T-cell lymphoma after the adoptive transfer of p53 null haematopoietic stem cells (HSCs) transduced to overexpress it. This indicated the potential for increased Fbxo7 to be oncogenic in T cells [29]. Given these data, and its capacity to directly bind to Cdk6 and promote cyclin D3/Cdk6 complex formation [13], we reasoned that it would be an important factor in T-cell biology. We report here that loss of Fbxo7 expression in a mouse impairs both thymocyte development and T-cell function. We demonstrate that Fbxo7 expression has opposing roles in cell proliferation within the T-cell lineage at different stages, promoting proliferation of thymocytes within the thymus, but restraining proliferation of activated T cells in the periphery. This paradoxical activity of Fbxo7 indicates that the G1 phase circuitry during T-cell development is differentially regulated from that of mature T cells.

## Materials and methods

### Mice

All experimental animals were maintained in accordance with animal licences approved by the Home Office and the University of Cambridge’s Animal Welfare and Ethical Review Body Standing Committee, and the ARRIVE guidelines. All work described here was performed under the Home Office licences PPL 80/2474 (expired 2016) and PPL70/9001 (valid until 2021). Fbxo7^LacZ^ mice (Fbxo7^tm1a(EUCOMM)Hmgu^ C57BL/6J background) were maintained in individually ventilated cages with unrestricted access to food and water, and heterozygous animals were bred. WT and homozygous littermates were harvested between 6–8 weeks, unless stated otherwise. Male and female mice were both used for experiments.

For genotyping, crude genomic DNA extraction was performed on ear punch biopsies. Tissue was digested using a solution of 10% Chelex (BioRad), 100 µg/mL proteinase K, and 0.1% Tween-20. One microliter of supernatant was used in multiplex genotyping, with a common 5′ forward primer (CAGGATCAGGGAACGCCTGT) and different 3′ reverse primers to amplify the WT (TGCAGGGTGAATAGCACTTCC) or the transgenic (CACAACGGGTTCTTCTGTTAGTCC) allele. The reaction amplifying the WT allele produces a 197 bp product, whereas the reaction amplifying the transgene produces a 362 bp product. The PCR consisted of 35 cycles of 92, 62, and 72 °C for 30 s each. Primers pairs spanning an exon–exon boundary were used for qRT-PCR of murine Fbxo7 using SYBR Green JumpStart Taq Ready Mix (Sigma) on an iCycler thermocycler (BioRad). Primers used were 5′ (CGCAGCCAAAGTGTACAAAG) and 3′ (AGGTTCAGTACTTGCCGTGTG).

### Tissue preparation

Spleens and thymuses were harvested and disaggregated by passage through a 40 μm cell strainer, and splenocytes subjected to RBC ammonium chloride lysis buffer to remove excess RBCs. Cell density was determined by trypan blue (Invitrogen) exclusion using a haemocytometer.

### Flow cytometry and fluorescence activated cell sorting (FACS)

Single cell suspensions of splenocytes and thymocytes were stained with fluorescently labelled CD4-PE or CD4-PECy7 (clone GK1.5) and CD8-APC (clone H35-17.2) antibodies, for 30 min at 4 °C in the dark, washed twice with FACS buffer (PBS with 1% FBS), and CD4^+^ or CD8^+^ positive cells analysed using Summit 4.3 software (Beckman Coulter) on a Cyan ADP Analyser (Dako), or sorted in PBS using a MoFlo FACS sorter (Dako). Sorted cells were resuspended in the required media for culturing, or lysed for immunoblotting. Cells were analysed using the following gating strategy for thymocytes and splenocytes (Supplementary Figs. 1A and B, respectively). Thymocytes were also stained with antibodies to CD4-biotin and CD8-biotin followed by Streptavidin-FITC, as well as antibodies to CD44-PE (clone IM7), CD25-PECy7 (clone PC61.5), and analysed or sorted using the following gating strategy (Supplementary Fig. 1C). Collected cells were used for cell cycle analysis or immunoblotting. All flow cytometry antibodies were from eBiosciences. For T_Reg_ cells, splenocytes were stained for CD3 and CD4, before being fixed, permeabilised, and subjected to intracellular Foxp3 staining following the manufacturer’s recommendations (eBioscience). Apoptosis was assayed by first staining cells with antibodies to detect specific populations, washed once in Annexin V binding buffer (10 mM HEPES, 140 mM NaCl, 2.5 mM CaCl_2_, pH7.4), and then stained for Annexin V^+^ cells with Annexin V-Alexa Fluor 647, as per the manufacturer’s recommendations (Catalogue no. A23204, Invitrogen). Propidium iodide (PI) was also used at 2.5 μg/mL to discriminate live cells from dead cells.

### T-cell activation and cytokine analysis

For T-cell activation assays, lymphocytes in mixed splenocyte suspensions were counted using a haemocytometer and 2 × 10^5^ cells plated per 96 well, in complete RPMI media (RPMI with 10% heat inactivated (HI) foetal bovine serum (FBS), 100 U/mL penicillin and streptomycin, and 5 µM 2-mercaptoethanol (all Invitrogen)) supplemented with or without soluble 2 µg/mL anti-CD3e (clone 145-2C11) and 2 µg/mL anti-CD28 (clone 37.51), to activate T cells. Cells were incubated for 24, 48, and 72 h in a humidified 37 °C, 5% CO_2_ incubator. To assay for activation markers, cells were analysed by flow cytometry with antibodies to CD4-PE, CD8-APC, CD25-PECy7, and CD69-FITC (clone H1.2F3, eBiosciences). See Supplementary Fig. 1d and e for representative FACS plots. For cytokine analysis, CD4^+^ T cell from spleen were FACS sorted and plated as above. Cells were incubated for 24 and 48 h, and then supernatants collected for cytokine analysis using ELISA assays for IL-2, IFN-γ, as per the manufacturer’s instructions (eBioscience).

### Cell cycle analysis

For carboxy fluorescein succinimidyl ester (CFSE) staining, splenocytes (3 × 10^6^ cells/mL) were incubated with 1 µM CFSE (Invitrogen) in PBS for 15 min in a humidified 37 °C, 5% CO_2_ incubator. Cells were washed twice with PBS supplemented with 10% FBS and resuspended in complete RPMI media. CFSE labelled cells (2 × 10^5^/well in 96-well plates) were seeded in a final volume of 200 µL complete RPMI media with soluble anti-CD3 and anti-CD28 as described above, and cultured for 48 and 72 h. Cells were analysed for CFSE dilution using flow cytometry. For cell cycle analysis using PI, thymocytes were FACS sorted, and stained as described in [15], and analysed by flow cytometry.

### Statistical analyses

Statistical differences between mutant and WT were calculated using Student’s two-tailed *t* tests with a significant cutoff of *p* < 0.05. Data are presented as mean ± standard deviation. On graphs, *p < 0.05, **p < 0.01, ***p < 0.001.

### Cell lysis

Sorted cells or whole thymuses were lysed in RIPA buffer (50 mM Tris–HCl pH 7.6, 150 mM NaCl, 1% NP-40, 0.1% SDS, 0.1% Na deoxycholate, 1× protease inhibitors, 1 mM PMSF, 10 mM sodium fluoride, 1 mM sodium orthovanadate) (all from Sigma), and incubated on ice for 30 min with occasional vortexing. Cell debris was pelleted by centrifugation at 16,000*g* for 10 min at 4 °C. Cell lysates were collected and protein concentration determined using the BCA method in a 96-well plate (Pierce). Protein samples were diluted in lysis buffer to the desired concentration, ensuring equal protein concentrations across all samples.

### Immunoblotting

Protein samples were mixed with equal volumes of 2× Laemmli buffer and denatured by incubating at 95 °C for 5 min. Proteins were then separated using Tris–Glycine SDS polyacrylamide gel electrophoresis (SDS-PAGE), and transferred onto polyvinylidene fluoride (PVDF) membrane (Millipore) using a semi-dry transfer system (Biorad). Membranes were blocked for 1 h with 5% non-fat, milk powder/PBS-Tween 20 (0.05%) (PBS-T), and then probed with primary antibody overnight at 4 °C in 5% non-fat, milk powder/PBS-T. Membranes were washed in PBS-T and incubated in the appropriate HRP-conjugated secondary antibody in 5% non-fat, milk powder/PBS-T followed by further washes, and detection of HRP bound protein using enhanced chemiluminescence (ECL, GE Healthcare) and exposure onto X-ray film (Konica Minolta). Signal was quantified and normalised using the ImageJ software (NIH, Maryland).

### Antibodies

Antibodies for western blotting were used as described in [16], as well as anti-cleaved caspase 3 (9664S), anti-phospho pRb Ser780 (8180S), and anti-phospho pRb Ser807/811 (9308S), all from Cell Signalling Technologies, and anti-GAPDH (G9545) and anti-γ-tubulin (T6557) both from Sigma-Aldrich.

## Results

### Defective thymocyte development in *Fbxo7*^*LacZ/LacZ*^ mutant mice

**Fig. 1:**
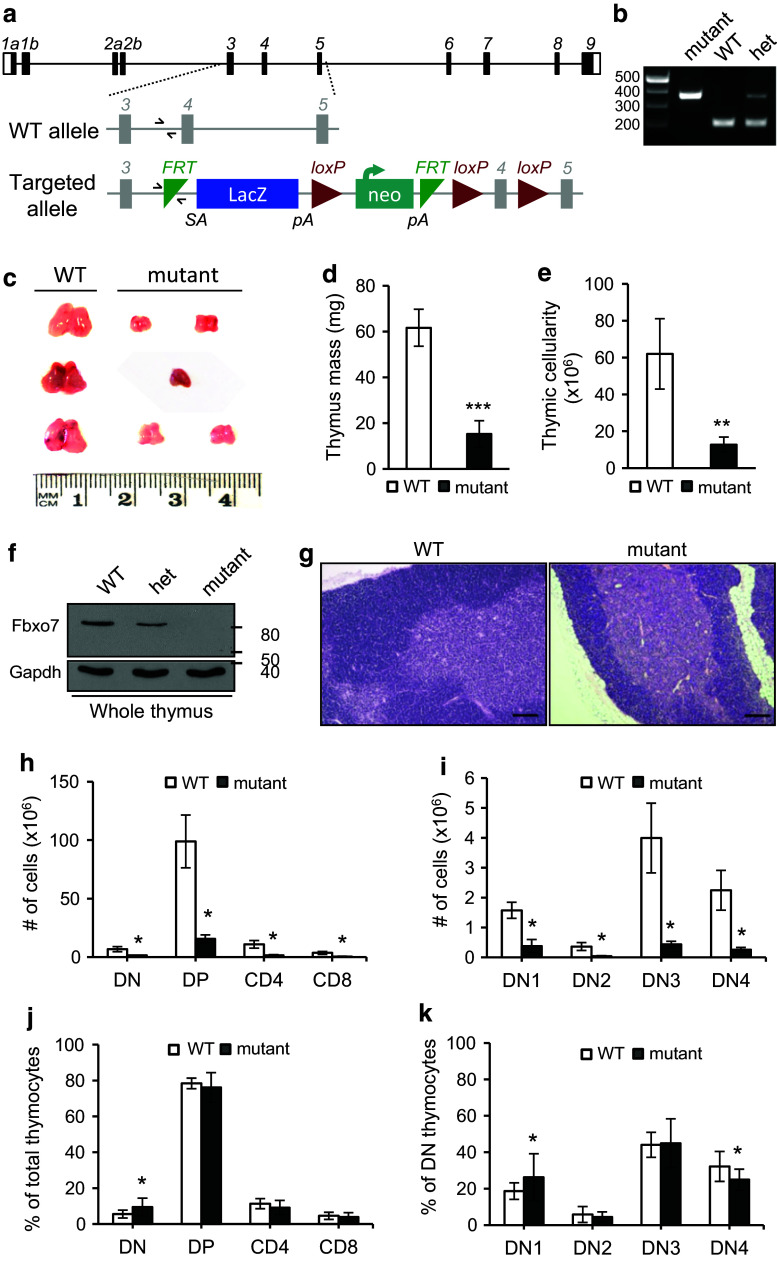
*Fbxo7* ^*LacZ/LacZ*^ mice have thymic hypoplasia. **a** Schematic of murine *Fbxo7* locus with WT and targeted allele, Fbxo7^tm1a(EUCOMM)Hmgu^, showing LacZ/neo insertion with splice acceptor (SA) site and poly-A tails (pA). Primers used for multiplex genotyping are shown (*half arrows*). **b** Multiplex PCR reaction showing amplification of WT (197 bp) and mutant (362 bp) alleles in WT, heterozygous (het), and homozygous LacZ (mutant) mice. The same forward primer is used in both reactions. **c** Photograph of thymuses from 2- to 4-month-old littermate WT and *Fbxo7* ^*LacZ/LacZ*^ mutant mice, from 3 L. **d** Quantification of thymus weight from 2- to 4-month-old mice (*n* = 4 WT and *n* = 8 mutant mice from 4 L). **e** Total thymic cellularity of WT (*n* = 6) and *Fbxo7* ^*LacZ/LacZ*^ (*n* = 5). **f** Lysates made from thymuses from WT, heterozygous, and homozygous Fbxo7^LacZ^ mice were analysed by immunoblotting for Fbxo7 and actin (*n* = 1). **g** H&E-stained sections of WT and *Fbxo7* ^*LacZ/LacZ*^ thymus showing medullary (*pink*) and cortical (*blue*) compartments. *Scale bars* 200 μm (*n* = 3 from 2 L). **h** Total cell number of indicated populations in WT (*n* = 6) and *Fbxo7* ^*LacZ/LacZ*^ (*n* = 5) thymuses. **i** Total cell number at each DN stage in WT (*n* = 7) and *Fbxo7* ^*LacZ/LacZ*^ (*n* = 6) thymuses. **j**, **k** Graphs showing percentages of cells in each thymocyte subpopulation from WT (*n* = 21) and *Fbxo7* ^*LacZ/LacZ*^ (*n* = 18) thymuses. Data are represented as mean ± SD, *p < 0.05, **p < 0.01, ***p < 0.001

The generation of Fbxo7^LacZ^ mice has been described previously [16], and a schematic of the transgene is shown in Fig. 1a. Expression of Fbxo7 is disrupted by the presence of the *LacZ* gene, with a preceding splice acceptor site, between exons 3 and 4 of *Fbxo7*. The identification of mice with the LacZ insertion was confirmed by multiplex PCR (Fig. 1b) where a common forward primer (half arrows, Fig. 1a) amplifies a 197 base pair product with a reverse primer to WT genomic sequences, or a 362 base pair product with a reverse primer to sequences in the transgene. Mice with homozygous inheritance of an Fbxo7^*LacZ*^ (mutant) allele consistently had visibly smaller thymuses (*n* = 45) compared to WT mice (Fig. 1c), which were on average 25% of the mass of WT (Fig. 1d). This phenotype was fully penetrant, and this was quantified as an 80% reduction in cell number (Fig. 1e). Homozygous Fbxo7^LacZ^ mice have approximately 80–90% reduction in Fbxo7 mRNA expression in many tissues, such as liver, spleen, and cerebellum, as shown by qRT-PCR analysis [15]. In the thymus, no Fbxo7 message is detected by qRT-PCR analysis [15], and we could not detect any protein expression by immunoblotting of *Fbxo7* ^*LacZ*/*LacZ*^ lysates from whole thymuses (Fig. 1f). Haematoxylin and eosin staining of thymuses harvested from littermate-matched WT and *Fbxo7* ^*LacZ*/*LacZ*^ mice showed intact medulla and cortical thymic compartments with no gross changes in their anatomical structure (Fig. 1g), arguing against premature involution as a cause of hypoplasia.

To investigate the cellular deficiencies in the thymus, the total cell numbers at all T-cell developmental stages were quantified. Single cell suspensions from thymuses from WT and *Fbxo7* ^*LacZ*/*LacZ*^ littermates were stained for CD4 and CD8 (double negative (DN), double positive (DP), and single positive either for CD4^+^ or CD8^+^) (Fig. 1h), or for CD25 and CD44 to identify developmental stages within the CD4 and CD8 DN population [CD25^−^ CD44^+^ (DN1), CD25^+^ CD44^+^ (DN2), CD25^+^ CD44^−^ (DN3), and CD25^−^ CD44^−^ (DN4)] (Fig. 1i). The absolute cell numbers were significantly reduced at all developmental stages (Fig. 1h, i). The relative proportions at each stage of T-cell differentiation were also determined, and we observed a statistically significant 0.73-fold increase in the total DN fraction (Fig. 1j), such that this fraction accounted for 9.5% of total thymocytes in mutant mice compared to 5.5% in WT mice. No significant differences were seen in the proportions of the DP or SP populations (Fig. 1j). To investigate whether a particular DN stage was affected, the proportion of cells at each DN stage in thymuses from mutant and WT mice were analysed. The percentage of DN1 cells was significantly increased in mutants compared to WT (0.41-fold increase), whereas the percentage of DN4 cells was reduced significantly (0.22-fold decrease) in mutants compared to WT (Fig. 1k). This proportional increase in DN1 cells in the mutant mouse must contribute to the statistically significant increase in the DN populations compared to DP and SP populations (Fig. 1j), since the DN2/DN3 proportions are similar and DN4 is reduced in the mutant thymuses (Fig. 1k). Thus, among DN populations in *Fbxo7* ^*LacZ/LacZ*^ mice, there were increased DN1 cells and fewer DN4, which may stem from changes in proliferation and/or apoptosis. We noted that when thymuses from mice older than 10 weeks were analysed, the proportions at individual DN stages were unchanged (Supplementary Fig. 2a), indicating that these defects can be compensated for over time.

### Reduced numbers of the early thymic progenitors (ETPs) in mutant mice

**Fig. 2:**
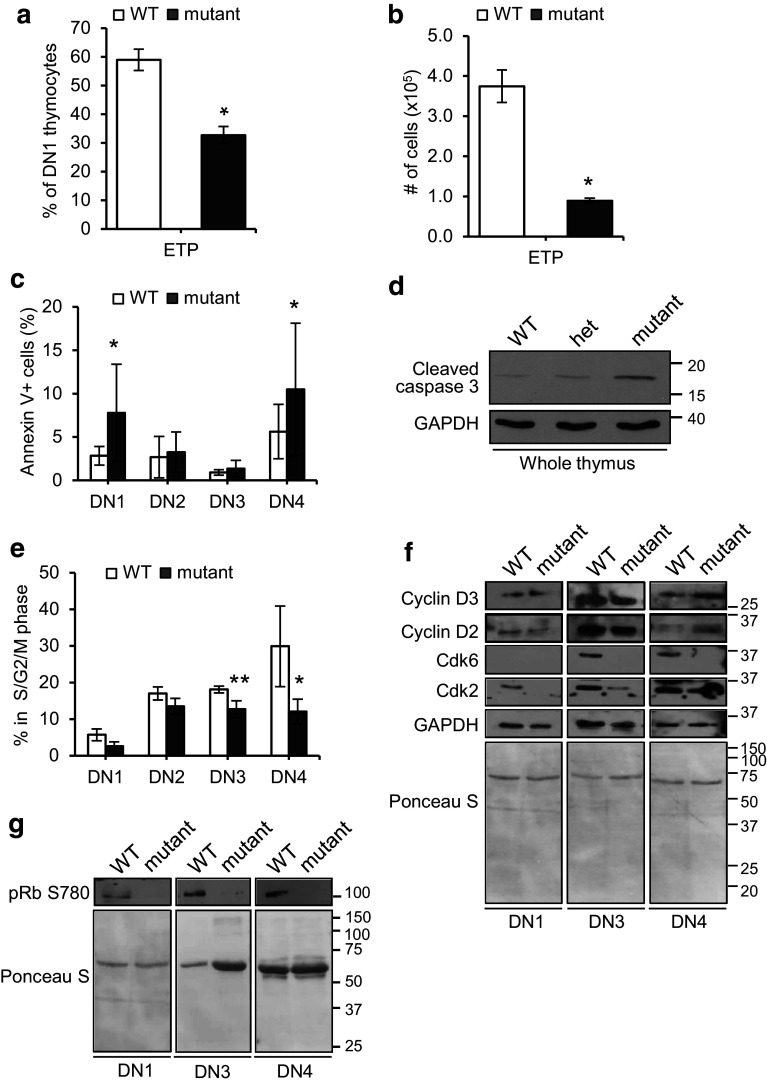
Decreased proliferation and increased apoptosis in the thymuses of *Fbxo7* ^*LacZ/LacZ*^ mice. **a**, **b** Graphs showing ETPs as a percentage of DN1 population (**a**) and as total number in thymuses (**b**) in WT (*n* = 4) and *Fbxo7* ^*LacZ/LacZ*^ (*n* = 4) thymuses. **c** Annexin V staining of DN1-4 populations from WT (*n* = 13) and *Fbxo7* ^*LacZ/LacZ*^ (*n* = 12) thymuses. Data represented as mean ± SD, *p < 0.05. **d** Immunoblotting for cleaved caspase 3 of lysates made from whole thymuses from animals of the indicated genotypes (*n* = 1). **e** Graph showing percentage of cells in S/G_2_/M phases of the cell cycle in each FACS sorted population from WT and *Fbxo7* ^*LacZ/LacZ*^ thymuses pooled from 15 mice. Data show mean of four independent experiments. *p < 0.05, **p < 0.01. **f**, **g** Representative western blot of lysates from FACS sorted DN1, DN3, and DN4 thymocytes, from four pooled WT and *Fbxo7* ^*LacZ/LacZ*^ thymuses, showing cell cycle regulatory proteins (**f**), or phosphorylated pRb (**g**). GAPDH and Ponceau S staining were used as a protein loading control as indicated. These experiments were repeated twice

A possible cause for thymic hypoplasia might be insufficient seeding of the thymus or defects in the survival or development of ETPs after seeding. To address this, we quantified, by flow cytometry, the number and proportion of bone marrow (BM)-derived ETPs, identified as c-Kit^+^ within the DN1 population (CD44^+^ CD25^−^ CD4^−^ and CD8^−^) cells. A significant reduction in the percentage (0.45-fold) and absolute number (0.76-fold) of ETPs was observed in *Fbxo7* ^*LacZ/LacZ*^ mice compared to WT (Fig. 2a, b). Although these data might suggest a BM insufficiency, a previous analysis of haematopoietic progenitor populations in the BM of *Fbxo7* ^*LacZ/LacZ*^ mice showed no significant changes in the percentages of LMPPs (Lin^−^ Sca-1^+^ c-Kit^+^ CD34^+^ and Flt3^+^) or CLPs (Lin^−^ IL-7Rα^+^ and Flt3^+^) compared to WT [15]. These data argue against a depleted progenitor pool in the BM as a cause of hypoplasia, but demonstrate the presence of fewer ETP cells in the thymus of mutant mice. This might occur due to reduced seeding of the thymus or decreased viability of ETPs within the thymus.

### Increased apoptosis and reduced proliferation of mutant DN thymocytes

We next investigated whether changes in the rates of apoptosis and/or proliferation within thymic subsets might account for the observed differences in mutant mice. To test this, thymocytes at all developmental stages were stained for Annexin V (Fig. 2c). Flow cytometry analysis showed a 1.74- and 0.87-fold increase in Annexin V staining at the DN1 and DN4 stages, respectively, in mutant cells compared to WT. There were no significant differences in Annexin V^+^ cells at the other DP, CD4 SP, and CD8 SP stages (Supplementary Fig. 2b). To further test this finding, immunoblotting of whole cell extracts of thymuses for cleaved caspase 3 showed that mice homozygous for the *Fbxo7* ^*LacZ*^ allele had the highest levels (Fig. 2d), supporting the idea that apoptosis levels increased when Fbxo7 expression was reduced.

We also tested whether the proliferation of thymocytes was altered. As an indicator of cell cycle phase, the DNA content was analysed by staining sorted DN populations with PI. Cells in G_0_/G_1_ phase (2 N DNA) and S/G_2_/M phases (>2 N DNA) were quantified and expressed as a percentage for each DN population. There was no statistically significant change in the percentage of cycling cells at the DN1 and DN2 stages. However, cycling cells (with >2 N DNA content) were significantly reduced by 29.5% in the DN3 population and by 59.5% in the DN4 population in mutant mice compared to WT (Fig. 2e). These data indicated that Fbxo7 is important for the proliferative burst that occurs at the DN3 and DN4 stages. Taken together, these data indicate that Fbxo7 loss causes deficiencies at multiple stages, including at the earliest DN1 stage, where we observed reduced ETP numbers and increased apoptosis, reduced proliferation at the DN3 stage, and both increased apoptosis and reduced proliferation at the DN4 stage in mutant mice.

### Lack of Cdk6 expression in *Fbxo7*^*LacZ/LacZ*^ thymocytes

Fbxo7 promotes the assembly of D-type cyclins with Cdk6 [13, 14], and thymocyte proliferation has been shown to be crucially dependent on Cdk6 [25, 26, 30]. To determine whether the absence of Fbxo7 affected the expression of these G1 cell cycle regulators, DN1/3/4 thymocytes from WT and *Fbxo7* ^*LacZ/LacZ*^ mutant mice were harvested, and cell lysates analysed by immunoblotting (Fig. 2f). The numbers of DN2 cells recovered were insufficient for analysis, due to the reduced cellularity of mutant thymuses and small percentage of DN2 cells. The most obvious difference seen was that Cdk6 was undetectable in samples from mutant mice, and there was also a reduction in Cdk2 levels compared to WT samples. In addition, in mutant DN4 thymocytes, there were increased levels of cyclins D2 and D3 compared to WT, but in DN1 and DN3 cells, D-type cyclin levels were similar (Fig. 2f).

To further investigate the molecular effect of the loss of Cdk6, we tested for its activity in DN populations by immunoblotting using phospho-specific antibodies for pRb phosphorylation at Ser780, as modification at this residue is used as a marker of cyclin D/Cdk6 activity. Equal numbers of DN1/3/4 cells from WT and mutant mice were harvested, and cell lysates were immunoblotted. All DN thymocytes from WT mice had readily detectable phospho-Ser780 pRb in contrast to DN thymocytes from mutant mice where none was detected (Fig. 2g). These data indicate that Cdk6 activity was absent in the tested DN populations of *Fbxo7* ^*LacZ/LacZ*^ mutant mice, and support the idea that Fbxo7 promotes cyclin D-Cdk6 activity, which promotes the proliferation of immature thymocytes.

### *Fbxo7*^*LacZ/LacZ*^ mice have fewer CD4^**+**^ and CD8^**+**^ T cells in the periphery

**Fig. 3:**
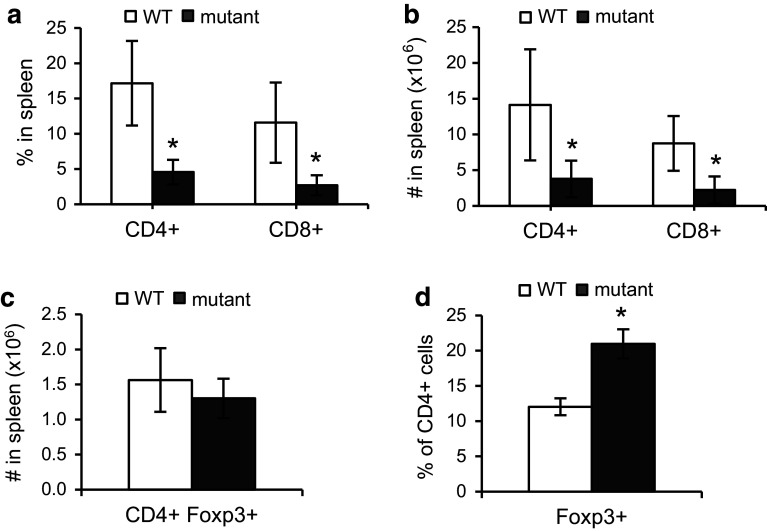
Decreased numbers of CD4 and CD8 T cells in the spleen of *Fbxo7* ^*LacZ/LacZ*^ mice. **a**, **b** Graphs showing the percentage (**a**) and absolute cell number (**b**) of CD4^+^ and CD8^+^ T cells in WT and *Fbxo7* ^*LacZ/LacZ*^ spleens (*n* = 10). **c** Graph showing percentage of Foxp3^+^ T_Reg_ cells in CD4^+^ population in spleen (*n* = 5). **d** Total number of CD4^+^ Foxp3^+^ T_Reg_ in WT and *Fbxo7* ^*LacZ/LacZ*^ mutant spleens (*n* = 5). All data are represented as mean ± SD, *p < 0.05

A lack of Fbxo7 expression resulted in significantly fewer cells differentiating to the SP stages within the thymus (Fig. 1h). To assess the effect on mature T cells in the periphery, the percentage and absolute numbers of peripheral CD4^+^ and CD8^+^ T cells were determined by counting T cells in the spleen. *Fbxo7* ^*LacZ/LacZ*^ mice have significantly enlarged spleens due to extramedullary haematopoiesis; however, splenic cellularity of mutant mice was comparable to WT littermates after the lysis of RBCs, indicating that the increase is due mostly to erythroid lineage cells [15]. Mature T cells were stained using antibodies to CD4 and CD8 surface markers and analysed by flow cytometry. There were 0.74- and 0.77-fold reductions in the proportions of CD4^+^ and CD8^+^ T cells in spleens from mutant mice compared to WT (Fig. 3a). The absolute number of CD4^+^ and CD8^+^ T cells was also significantly reduced, in mutant mice (Fig. 3b). These data showed a significant reduction in peripheral CD4^+^ and CD8^+^ effector T cells of *Fbxo7* ^*LacZ/LacZ*^ mice, consistent with the reduced cellularity observed in the thymus.

Within the CD4 population, we also tested whether mutant mice had altered numbers of Foxp3^**+**^ regulatory T cells (T_Reg_) which suppress the immune response. Splenocytes were harvested and stained for CD4, CD25, and Foxp3. Although the number of CD4^**+**^ Foxp3^**+**^ cells was the not significantly different between WT and mutant mice, proportionally, there was a statistically significant 0.75-fold increase in CD4^+^ Foxp3^+^ T_Reg_ cells, due to the overall decrease in effector T cells in mutant mice (Fig. 3c, d).

### CD4^**+**^ and CD8^**+**^ T cells lacking Fbxo7 showed delayed activation

**Fig. 4:**
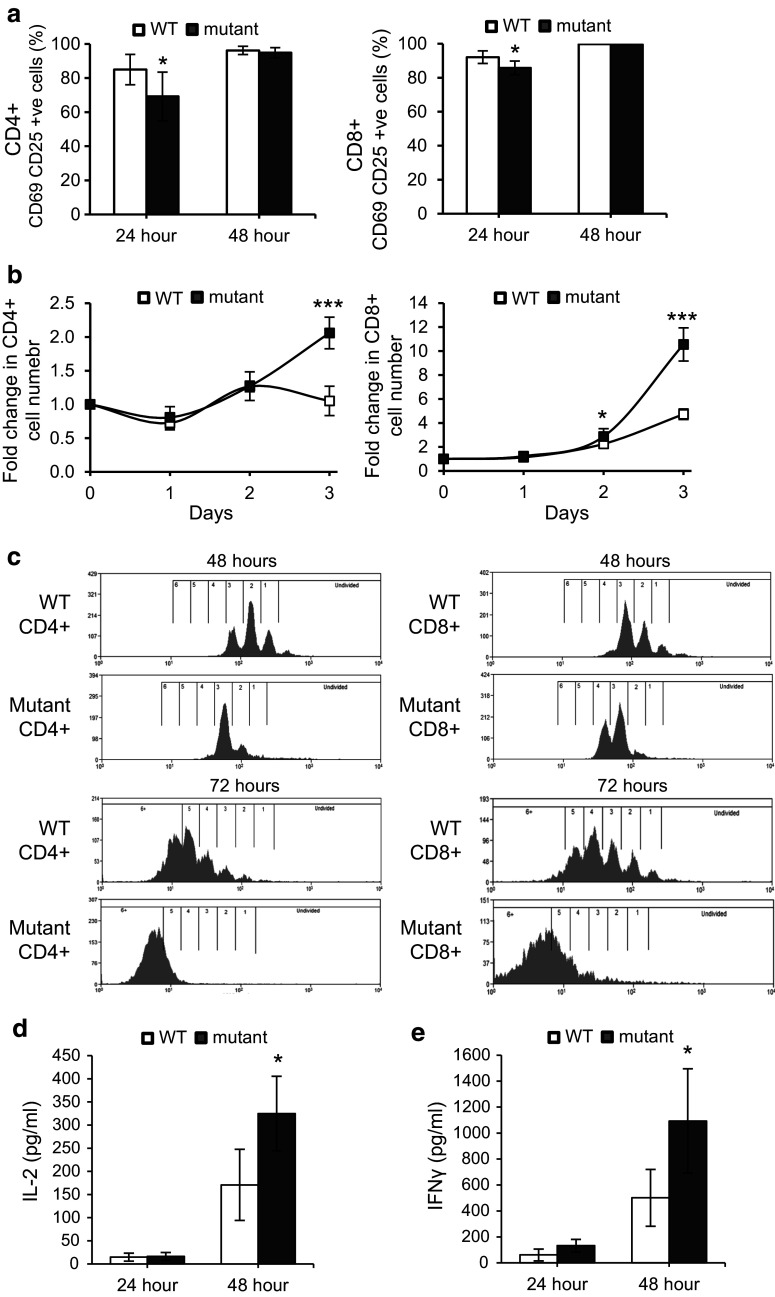
Delayed activation and more rapid expansion of mutant splenic CD4^+^ and CD8^+^ T cells. **a** Graph showing the percentage of CD69 and CD25 double positive CD4^+^ (*left*) and CD8^+^ (*right*) T cells in stimulated WT and *Fbxo7* ^*LacZ/LacZ*^ spleen cultures at 24 and 48 h post-stimulation (*n* = 10). **b** Graphs showing fold expansion in WT and *Fbxo7* ^*LacZ/LacZ*^ CD4^+^ (*left*) and CD8^+^ (*right*) T-cell numbers, normalised to day 0, at indicated time points in spleen cultures after CD3 and CD28 stimulation (*n* = 5). **c** Representative histograms displaying CFSE dilution of WT and *Fbxo7* ^*LacZ/LacZ*^ CD4^+^ T (*left*) and CD8^+^ (*right*) T cells at 48 (*top two histograms*) and 72 (*bottom two histograms*) h after TCR engagement (*n* = 7). **d**, **e** Graphs showing the levels of IL-2 (**d**) and IFN-γ (**e**) from FACS sorted WT and *Fbxo7* ^*LacZ/LacZ*^ CD4^+^ T cells cultures after 24 and 48 h of TCR stimulation with CD3 and CD28 (*n* = 5). For all experiments, data represented as mean ± SD. *p < 0.05, ***p < 0.001

To assess the role of Fbxo7 in T-cell activation in the mature CD4^+^ and CD8^+^ T-cell populations, we assayed for the expression of surface markers CD69 and CD25 after TCR stimulation with soluble antibodies to CD3 and CD28. Equal numbers of T cells were analysed, and both CD4^+^ and CD8^+^ T-cell populations from mutant mice showed significantly fewer double positive cells (16% fewer for CD4^+^ and 6% for CD8^+^ T cells) compared to WT at 24 h after TCR stimulation, (Fig. 4a, Supplementary Fig. 1d, e). At 48 h post-stimulation, the percentages of WT and mutant CD4^+^ and CD8^+^ T cells expressing both activation markers were comparable. In the absence of stimulation by CD3/CD28, no CD25^+^ CD69^+^ cells were detected. Thus, both CD4^+^ and CD8^+^ T cells show delayed activation in the absence of Fbxo7, indicating a requirement for Fbxo7 function in the timely activation of T cells.

### Fbxo7-deficient T cells expand more rapidly after TCR engagement

T-cell activation is followed by rapid increases in cell number, with cell doubling times reported to be as little as 4.5 h [31], and differentiation into effector T cells. We next tested whether proliferation was altered in mutant cells. Splenocytes were stimulated with soluble antibodies to CD3 and CD28, and the percentages of CD4^+^ and CD8^+^ T cells were monitored over 3 days. At the outset, CD4^+^ T cells comprised 16.4% of WT and 5.9% of mutant splenocyte cultures (Supplementary Fig. 2c). After 24 h, this number was largely unchanged in both cultures. However, by 72 h, the percentage of CD4^+^ T cells in WT cultures was 13.1% and comparable to the percentage of CD4 cells in mutant cultures of 11.4%. When the fold change in absolute cell number was calculated, mutant CD4^+^ T cells had expanded by 1.27- and 2.06-fold at 48 and 72 h compared to the starting number, whereas WT CD4^**+**^ T cells expanded 1.27 fold by 48 h but was back to day 0 numbers by 72 h (Fig. 4b, left). Thus, the significant difference between the numbers of WT and mutant CD4 cells observed at the start of the assay was lost by 72 h due to the more rapid expansion of mutant CD4^+^ cells (Supplementary Fig. 2c).

A similar analysis was performed for CD8^+^ T cells. At day 0, there were significantly fewer CD8^+^ T cells in mutant cultures: 2.6% compared to 9.2% in WT samples (Supplementary Fig. 2d). By 72 h, *Fbxo7* ^*LacZ/LacZ*^ and WT cultures contained 25.9 and 34.7% CD8^+^ T cells, respectively, showing that both WT and *Fbxo7* ^*LacZ/LacZ*^ CD8 cells expanded robustly. However, when the fold change in absolute cell number was calculated, *Fbxo7* ^*LacZ/LacZ*^ cultures had expanded to a much greater extent over 72 h (Fig. 4b, right). At 48 and 72 h, WT cells expanded 2.3- and 4.7-fold compared to 2.9- and 10.6-fold expansion of mutant cells at the same time points. Notably, despite their greater expansion, quantification of the absolute CD4 and CD8 cell number in activated cultures over the course of the experiment showed that the number of mutant T cells did not exceed that of WT T cells (Supplementary Fig. 2e, f), These data demonstrated that activated CD4^+^ and CD8^+^ T cells lacking Fbxo7 increased in numbers significantly more than WT cells.

### *Fbxo7*^*LacZ/LacZ*^ T cells cycle more rapidly after activation

To investigate the observed greater increase in the numbers of mutant T cells following activation, we tested whether activated T cells showed changes in their rates of proliferation. After treatment with RBC lysis buffer, equal numbers of WT and mutant splenocytes were first labelled with a fluorescent dye CFSE and then incubated with CD3 and CD28 antibodies. Flow cytometry was used to monitor CFSE dilution as an indicator of cell divisions at 48 and 72 h post-activation in both CD4^+^ and CD8^+^ cells (Fig. 4c). At 48 h, 74.6% of mutant CD4^+^ T cells had undergone 3 or more cell divisions compared to 36.9% in WT samples (Fig. 4c, top row), and by 72 h, 77.7% of CD4^+^ mutant T cells had divided 5 or more times compared to 35.3% in WT cultures (Fig. 4c, bottom row). Similarly, at 48 h, 94.2% of CD8^+^ mutant T had divided 3 or more times compared to 57.8% in WT samples (Fig. 4c, top row), and by 72 h, 99.7% CD8^+^ mutant T cells had undergone 5 or more cell divisions compared to 83.1% in WT cultures (Fig. 4c, bottom row). These data show that after activation, Fbxo7 mutant T cells cycle significantly faster than WT cells, and indicate an anti-proliferative role for Fbxo7 in the proliferation of activated T cells.

### Increased IL-2 and IFN-γ production by *Fbxo7*^*LacZ/LacZ*^ CD4^+^ T cells

Upon TCR activation, naive CD4^+^ T cells may differentiate into one of several Th lineages, each defined by their pattern of cytokine production and function. We tested for Th1 and Th2 cytokines secreted by isolated WT and mutant CD4^+^ T cells after TCR activation with soluble antibodies to CD3 and CD28 molecules. At 24 and 48 h, after TCR activation, supernatants from T cells cultures were collected and cytokines concentrations were measured using ELISAs. Levels of Th1 and Th2 cytokines were assayed to give an overview of effector functions of CD4^+^ T cells. At 24 h, the IL-2 concentration in mutant CD4^+^ T samples was comparable to WT samples, and at 48 h, the IL-2 concentration was on average 325 pg/mL in mutant CD4^+^ T-cell samples compared to 171 pg/mL in WT samples, a 1.9-fold increase (Fig. 4d). Similarly, at 48 h post-stimulation, mutant CD4 cells produced 1093 pg/mL of IFN-γ compared to 501 pg/mL in WT cultures, representing a 2.2-fold increase, which was statistically significant (Fig. 4e). These results indicated that mutant CD4^+^ T cells produced a Th1 cytokine response similar to WT, suggesting that subsequent activation of other immune cells, such as macrophages, and CD8^+^ T cells would be possible in *Fbxo7* ^*LacZ/LacZ*^ mice. The levels of IL-4 and IL-10, indicative of a Th2 response, were also determined, and both were similar in WT and mutant samples (data not shown). These results showed that activated mutant CD4^+^ T cells were capable of producing both Th1 and Th2 cytokines. The approximate twofold increase in the production of IL-2 and IFN-γ despite overall fewer cell numbers may be a due to the more rapid proliferation of mutant CD4 cells upon activation.

### Increased apoptosis in resting and activated mutant CD4^**+**^ and CD8^**+**^ T cells

**Fig. 5:**
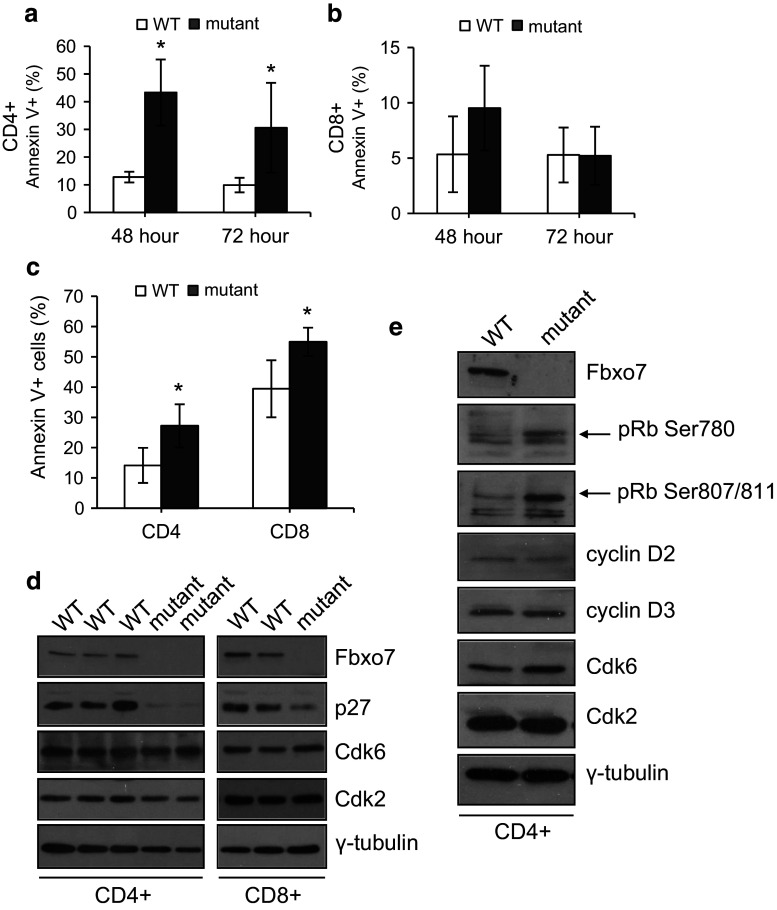
Decreased levels of p27 in mutant T cells. **a**, **b** Graphs showing the percentage of Annexin V positive cells in activated WT and mutant CD4^+^ (**a**) and CD8^+^ (**b**) T cells at 48 and 72 h after TCR stimulation of splenocyte cell cultures (*n* = 10). **c** Graph showing the percentage of Annexin V positive cells in naïve CD4^+^ and CD8^+^ T cells in WT and *Fbxo7* ^*LacZ/LacZ*^ spleens (*n* = 4). For all experiments, data represented as mean ± SD. *p < 0.05. **d**, **e** Representative images of western blot of lysates made from equal numbers of FACS sorted CD4^+^ and CD8^+^ T cells from WT and *Fbxo7* ^*LacZ/LacZ*^ spleens. Due to low numbers of mature T cells, mutant cells were pooled from 5 (**d**) or 4 (**e**) mutant mice. These experiments were repeated twice

Since mutant T cells expanded more rapidly than WT cells after activation, we tested whether the capacity to undergo apoptosis was inhibited which might also contribute to the observed increased expansion upon activation. To determine the percentage of T cells undergoing apoptosis, WT and mutant splenocytes were stimulated as above. At 48 and 72 h of TCR stimulation, cells were removed and co-stained with antibodies to CD4 and CD8 along with Annexin V (Fig. 5a, b). Interestingly, there were significantly more apoptotic mutant CD4^+^ T cells: 43.3% compared to 12.8% for WT cultures at 48 h, and 30.6% compared to 9.9% at 72 h, reflecting a 2.4 and 2.1 fold increase, respectively, in stimulated mutant CD4^+^ cells (Fig. 5a). In contrast, 48 h post-stimulation, 9.5% of mutant CD8^+^ T cells were Annexin V^**+**^ compared to 5.3% cells in WT samples, a difference which was not statistically significant (*P* = 0.051). At 72 h, both cultures had approximately 5% Annexin V^**+**^ cells (Fig. 5b). Thus, mutant CD4^+^ T cells, but not CD8^+^ T cells, showed significantly increased levels of apoptosis after activation, and demonstrate that apoptosis induction was not suppressed in mutant T cells. The higher rates of apoptosis in CD4^+^ compared to CD8^+^ cells (~13 vs. 5% for WT cultures) may contribute to the more limited expansion of CD4 cells relative to CD8 cells observed in culture (Supplementary Fig. 2e, f).

Resting T cells are thought to be more resistant to apoptosis than activated T cells. To investigate if increased apoptosis was also observed in resting T cells, unstimulated splenocytes from mutant and WT mice were also tested for staining with Annexin V (Fig. 5c). Flow cytometry analysis showed significantly greater numbers of Annexin V^+^ mutant CD4^+^ and CD8^+^ T cells. There was a 0.93-fold increase in the number of Annexin V^+^ cells: 27.2% mutant CD4^+^ cells compared to 14.1% in WT mice, and about a 0.4-fold increase in Annexin V^+^ mutant CD8^+^ cells: 54.9% compared to 39.4% in WT mice (Fig. 5c). These results indicated that resting T cells in the periphery of mutant mice undergo higher levels of apoptosis, which suggests that they may a have shorter lifespan. This may contribute to the decreased number of mature T cells present in the periphery, and suggests that Fbxo7 may regulate molecular pathways governing the survival of resting T cells.

### *Fbxo7*^*LacZ/LacZ*^ T cells have less p27

p27 is a critical mediator of T-cell quiescence, and TCR crosslinking causes both transcriptional and translational down-regulation of p27, leading to increased cyclin E/Cdk2 and A/Cdk2 activity, which promotes S phase entry [32]. We hypothesized that one mechanism to account for the more rapid cycling and increased apoptosis seen in mutant T cells might be reduced p27 levels. CD4^+^ and CD8^+^ T cells were isolated from spleens, and equal numbers of cells from each population were lysed. Protein lysates were immunoblotted, and both CD4^+^ and CD8^+^ mutant T cells showed reduced p27 levels (Fig. 5d). One consequence of reduced p27 expression would be a reduced inhibitory threshold for the activation of late G_1_ and S phase cyclin E/Cdk2 and A/Cdk2 activities. This would facilitate cell proliferation in activated T cells or directly induce apoptosis in quiescent cells, if appropriate secondary stimuli were not present. To test whether Cdk activity was increased in quiescent cells, phospho-specific antibodies to detect pRb modification were used to probe cell lysates from resting CD4^+^ T cells purified as in 5d. We observed increased signals for phosphorylation at Ser780 and at Ser807/811, indicating inactivation of pRb (Fig. 5e). Interestingly, Cdk6 expression was similar in mutant and WT cells, indicating that in peripheral T cells, Fbxo7 is not required to stabilize its expression or its activation by D-type cyclins. These data indicate that increased proliferation and apoptosis in *Fbxo7* ^*LacZ/LacZ*^ T cells may be explained by reduced p27 expression and increased overall Cdk activity, but not levels.

## Discussion

Our data on the defects within the T-cell lineage that arise due to the absence of Fbxo7 expression reveal its critical activities at different stages of development and function. Most striking is the fact that Fbxo7 has opposing roles in regulating cell cycle proliferation in a single cell type, stemming from its ability to act as a scaffold and stabilizer of the levels of Cdk6 and p27, whose functions appear to predominate at different times in T-cell biology [13–16]. During the development of T cells in the thymus, Fbxo7 appears to be required for proliferation at the DN3/DN4 stages, yet in the periphery, it functions to restrain proliferation after T-cell activation, as evidenced by the very rapid cell division of mature T cells after they are CD3/CD28 stimulated.

The roles of Cdk6 and p27 within the thymus have been independently tested using knock-out mouse models. The previously reported Cdk6 null mouse models both have reduced cellularity in their thymus [25, 30]. Deficiencies in cell number disproportionately decreased the immature thymocyte populations; however, small *increases* in thymic SP CD4^+^ and CD8^+^ cell numbers were observed. DN2 and DN3 cells showed reduced proliferation, and reduced apoptosis was observed at DN3, DN4, and SP cells. In the periphery, mature Cdk6 null T cells were slower to proliferate after mitogenic stimulation, although overall numbers of peripheral T lymphocytes were comparable to WT. These studies indicate that Cdk6 regulates the differentiation of thymocytes and the proliferative response of activated T lymphocytes. Fbxo7-deficient mice mimicked certain phenotypes of the Cdk6 null mouse, most obvious being that mutant DN cells showed no Cdk6 expression or any evidence of Cdk6 activity as shown by the absence of Ser780 pRb, which is the likely defect underlying the failure of DN3/DN4 cells to proliferate in mutant mice. Cyclin D3 KO mice also show proliferative defects at the DN3/DN4 stages [27]. Both cyclin partners D2 and D3 were detected in mutant mice, so the lack of Cdk6 was not due to their absence. Indeed, the inability to detect Cdk6 in mutant cells may be due to its inability to assemble with cyclin D2 and D3 in the absence of Fbxo7, which causes Cdk6 destabilisation. Cyclins D2 and D3 may interact with the other Cdks, 4 or 2, neither of which interact with Fbxo7 [13], but neither of which can substitute for Cdk6 activity at this critical stage in T-cell development [26, 30]. In contrast to the Cdk6 null and Fbxo7^LacZ^ mouse models, the three p27 KO mouse models demonstrate enlarged thymuses, with no alterations to T-cell development [33–35], suggesting that mis-regulation of p27 plays a minimal role in the thymic phenotype observed in Fbxo7^LacZ^ mice. An additional, unique defect we uncovered within the Fbxo7-deficient thymus was at the ETP/DN1 stage, where we observed decreased numbers of ETPs and also increased apoptosis of DN1 cells. These data indicated multiple defects in T-cell development at the earliest settling of the thymus by precursor cells, and their later proliferative expansion stages at the DN3/DN4 stages, along with increased apoptosis at the DN4 stage. The absence of Cdk6 at the DN3 and DN4 stages is likely to underlie the failure of these cells to expand; however, Cdk6 was not detected at the DN1 stage, and is, therefore, unlikely to account for the defects then.

Within the spleen, *Fbxo7* ^*LacZ/LacZ*^ mice had fewer mature CD4^+^ and CD8^+^ T cells, which displayed delayed activation, similar to Cdk6 null cells [25, 26], yet faster proliferation upon activation, which is likely to be due to decreased levels of p27 and the inactivation of pRb seen in these cells. Indeed, the latter phenotype is akin to that of p27 KO T cells, which have been shown to have increased numbers of cells in S phase, higher cyclin E activated kinase activity, and increased responsiveness to mitogens [33]. However, these cells also showed no changes in their levels of apoptosis, suggesting that their increased Cdk activity leads to increased cell proliferation and hyperplasia rather than cell death [33]. Both naïve CD4^+^ and CD8^+^ T cells and activated CD4^+^ T cells from Fbxo7-deficient animals showed increased levels of apoptosis, which suggests that aberrant cell signalling, rather than increased proliferation, is leading to increased cell death. Notably, since CD8^+^ T cells proliferate faster and more robustly than CD4^+^ T cells, yet do not show increased apoptosis levels, this argues against enhanced proliferation as the cause of apoptosis. An alternate possibility is that increased proliferation contributes to increased apoptosis, but mutant CD8^+^ T cells lacking Fbxo7 expression were protected from death due to the presence of growth factor(s) in the cultures providing survival signals. For instance, IL-2 secreted by activated CD4^+^ T cells and to lesser extent by activated CD8^+^ T cells is a key regulator of T-cell apoptosis. IL-2 levels together with the intensity of antigenic stimulation influence the viability of activated T cells [36]. A third possibility is that *Fbxo7* ^*LacZ/LacZ*^ CD8^+^ T cells may also undergo increased levels of apoptosis, but the effect is offset by their high proliferation rate.

Overall, *Fbxo7* ^*LacZ/LacZ*^ mice show a dramatically reduced number of peripheral T cells which show delayed activation. Moreover, within their significantly reduced CD4 T-cell population, they show a proportional increase in T_Reg_ cells, which may limit their immune responses. However, once activated both CD4 and CD8 cells proliferate robustly, despite the higher levels of apoptosis of CD4 cells. In addition, activated Fbxo7-deficient CD4^+^ cells were capable of producing both Th1 and Th2 response cytokines and produced ~twofold more IL-2 and IFN-γ. Collectively, these attributes suggest that these mice may be compromised in their immune responses. In support of this idea, we noted the results of a *Salmonella typhimurium* challenge using 6-week-old C57BL/6-Fbxo7^tm1a(EUCOMM)Wtsi^ mice performed by the Mouse Phenotyping Consortium. Their study showed that these mice had a significantly increased susceptibility to *Salmonella*-induced morbidity, with significantly higher bacterial counts in the spleen, liver, and caecal contents of Fbxo7-deficient mice compared to WT (Wellcome Trust Sanger Institute, Cambridge UK; personal communication) [37].

In addition to the extensive perturbations in the production and function of T cells caused by the loss of Fbxo7, our findings demonstrate the differential regulation of the G1 phase of the cell cycle in this cell lineage. These effects, which differ from the phenotypes of the single Cdk6 or p27 KO mouse models, reflect the capacity of Fbxo7 to act as a cell cycle regulator that directly impacts on both proteins that clearly provide non-redundant and critical functions at different stages in the development and functioning of T cells.

### Electronic supplementary material

Below is the link to the electronic supplementary material.

**Supplementary Fig.** **1.** (a–c) Gating strategies for flow cytometry used to detect CD4 and CD8 SP T cells in thymus (a) and spleen (b), as well as DN cells in thymus (c). (d, e) Representative flow cytometry plots showing the percentage of activated (double positive CD25^+^ , CD69^+^) CD4 (d) and CD8 (e) cells after stimulation with anti-CD3 and anti-CD28 antibodies, for 24 h (top panel) and 48 h (middle panel), or culture for 48 h with no activating antibodies (bottom panel)

**Supplementary Fig.** **2.** (a) Graph showing percentage of DN thymocyte population in mice older than 10 weeks in WT (n = 12) and *Fbxo7* ^*LacZ/LacZ*^ (n = 10) thymuses. (b) Annexin V staining of DP, CD4 and CD8 SP populations of WT (n = 6) and *Fbxo7* ^*LacZ/LacZ*^ (n = 5) thymuses at 6–8 weeks of age. (c, d) Graphs showing the percentage of CD4^+^ (c) and CD8^+^ (d) T cells in mixed splenocytes cultures from WT and *Fbxo7* ^*LacZ/LacZ*^ mutant mice, at the indicated time points after CD3 and CD28 stimulation (n = 5). (e, f) Graphs showing the number of CD4^+^ (e) and CD8^+^ (f) T cells in mixed splenocytes cultures from WT and *Fbxo7* ^*LacZ/LacZ*^ mutant mice, at the indicated time points after CD3 and CD28 stimulation (n = 5). Data represented as mean ± SD, ***p < 0.001

Supplementary material 1 (PPTX 609 kb)
