# Supplementary material for: Opposing effects on the cell cycle of T lymphocytes by Fbxo7 via Cdk6 and p27
Source: Cell Mol Life Sci. 2016 Dec 3;74(8):1553–66. doi: 10.1007/s00018-016-2427-3 (PMC5357273; doi:10.1007/s00018-016-2427-3)
Supplement: Supplementary file 1 — Supplementary material 1 (PPTX 609 kb) [file 18_2016_2427_MOESM1_ESM.pptx]

## Slide 1
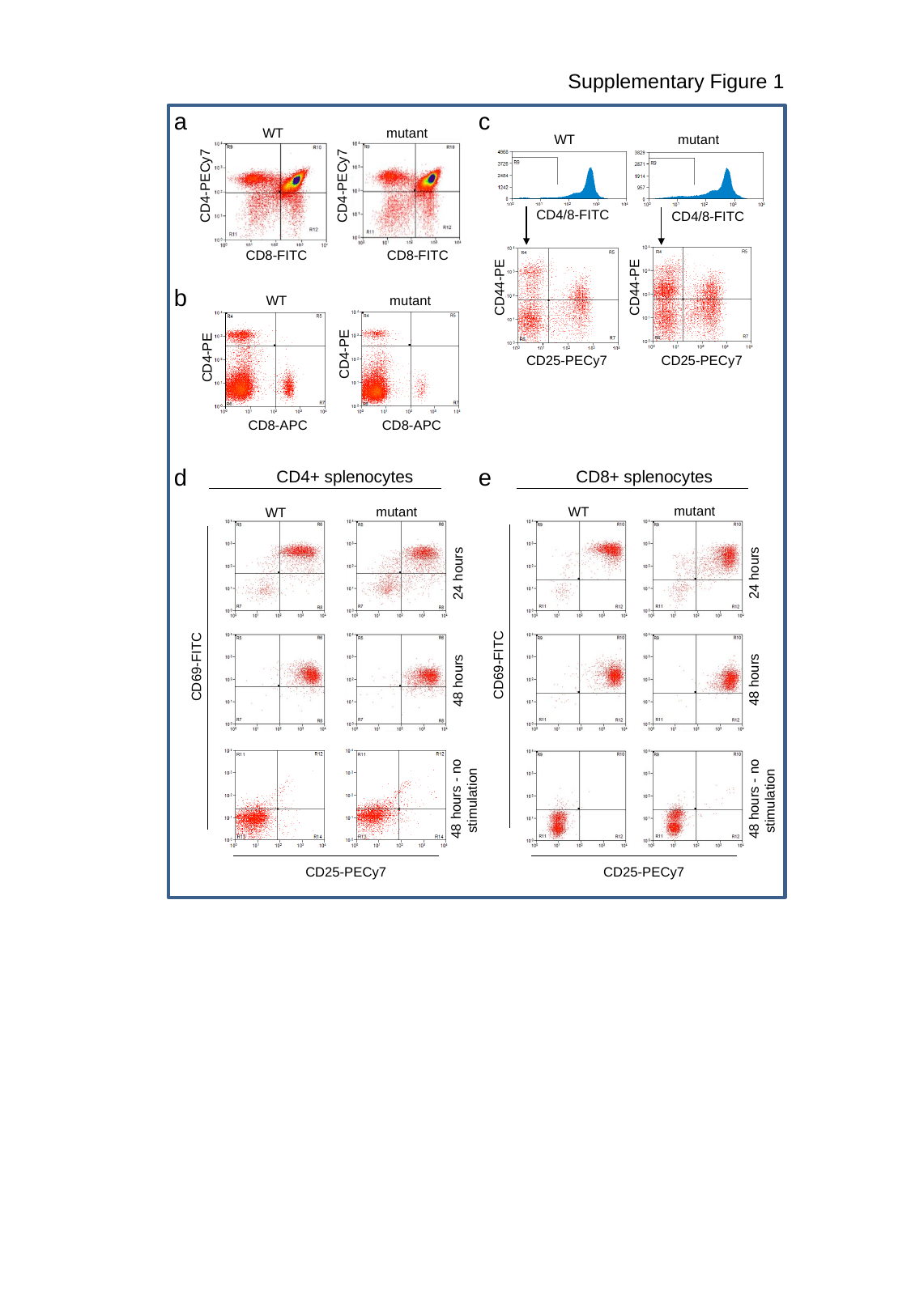

Supplementary Figure 1
a
c
WT
mutant
WT
mutant
CD4-PECy7
CD4-PECy7
CD4/8-FITC
CD4/8-FITC
CD8-FITC
CD8-FITC
CD44-PE
CD44-PE
b
WT
mutant
CD4-PE
CD4-PE
CD25-PECy7
CD25-PECy7
CD8-APC
CD8-APC
d
e
CD4+ splenocytes
CD8+ splenocytes
mutant
WT
mutant
WT
24 hours
24 hours
CD69-FITC
CD69-FITC
48 hours
48 hours
48 hours - no stimulation
48 hours - no stimulation
CD25-PECy7
CD25-PECy7

## Slide 2
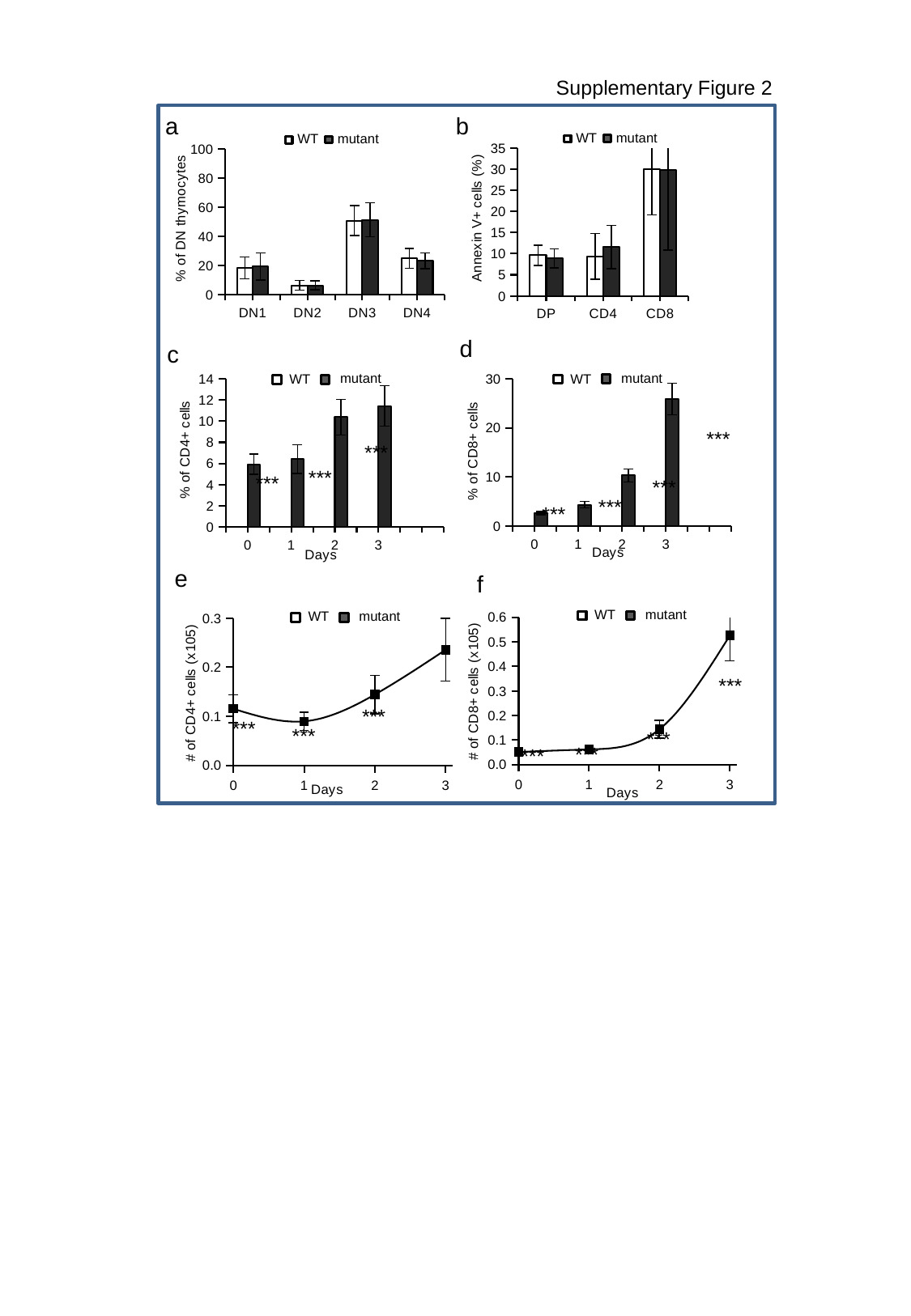

Supplementary Figure 2
a
b
### Chart
| Category | WT | Fbxo7LacZ/LacZ |
|---|---|---|
| DN1 | 18.382727272727266 | 19.361818181818187 |
| DN2 | 6.365454545454544 | 6.379090909090909 |
| DN3 | 50.88454545454544 | 51.4309090909091 |
| DN4 | 24.830909090909092 | 23.22636363636363 |mutant
WT
mutant
WT
### Chart
| Category | WT | Fbxo7 KO |
|---|---|---|
| DP | 9.633333333333333 | 8.864 |
| CD4 | 9.33666666666667 | 11.564000000000002 |
| CD8 | 29.951666666666668 | 29.826 |
### Chart
| Category | WT | mutant |
|---|---|---|
| 0 | 9.20133333333333 | 2.6366666666666663 |
| 1 | 12.920666666666671 | 4.356666666666667 |
| 2 | 18.92266666666666 | 10.338000000000001 |
| 3 | 34.64733333333333 | 25.889999999999993 |mutant
WT
***
***
***
***
d
### Chart
| Category | WT | mutant |
|---|---|---|
| 0 | 16.372 | 5.918666666666668 |
| 1 | 15.255333333333336 | 6.396666666666667 |
| 2 | 19.218666666666664 | 10.381333333333334 |
| 3 | 13.109333333333332 | 11.430666666666667 |c
mutant
WT
***
***
***
e
f
### Chart
| Category | WT | mutant |
|---|---|---|
### Chart
| Category | WT | mutant |
|---|---|---|mutant
WT
***
***
***
mutant
WT
***
***
***
***
